# Direct evidence of active tectonics along the offshore sector of the Dinaric Fault System

**DOI:** 10.1038/s41598-025-32243-z

**Published:** 2025-12-19

**Authors:** Tvrtko Korbar, Ozren Hasan, Dea Brunović, Snježana Markušić, Tiago Alves

**Affiliations:** 1 Croatian Geological Survey, Milana Sachsa 2, Zagreb, Croatia; 2 Department of Geophysics, Faculty of Science, University of Zagreb, Horvatovac 95, Zagreb, Croatia; 3 3D Seismic Lab, School of Earth and Environmental Sciences, Cardiff University, Main Building – Park Place, Cardiff, CF10 3AT UK

**Keywords:** Adriatic sea, Sub-bottom profiler, Quaternary deposits, Strike-slip faults, Seismotectonics, Natural hazards, Solid Earth sciences

## Abstract

Tectonic deformation along the External Dinarides fold-and-thrust belt is slow, with transpressional crustal strain redistributed along multiple faults. Some of these faults reach the surface along the NE Adriatic coast as part of the strike-slip Dinaric Fault System (DFS). This work uses new high-resolution sub-bottom seismic data to characterize the geodynamic significance of active surface deformation in the offshore sector of the Kvarner area, Croatia. Seismic profiles reveal gentle folding in early Quaternary strata that is associated with contractional deformation in the Rijeka Bay; yet, active faults are documented only in narrow zones along the DFS, and specifically in the Vinodol and Velebit channels. Fault Mechanism Solutions (FMS) confirm that a strike-slip tectonic regime exists in the study area, but the strike of surface faults and FMS data are discrepant, probably as a result of strain dissipation along (creeping? ) faults whose geodynamic response differs from deep-rooted seismogenic structures. This differing geodynamic response causes important caveats when linking surface deformation to deeper seismogenic structures which are, offshore Kvarner, either blind structures or currently deforming under distinct stress conditions to near-surface faults. Crucially, this work presents the first geological evidence for active faulting along the DFS in Croatia, a piece of information deemed critical for future geohazard assessments.

**Supplementary Information:**

The online version contains supplementary material available at 10.1038/s41598-025-32243-z.

## Introduction

Neotectonics, as a geological term, refers to the geodynamic phenomena that have occurred during the last few million years, while *active tectonics* considers those processes that are only active for the last ~ 100,000 years^1–4^. In practice, active tectonic movements are primarily identified via seismological data gathered during the rupture of an active fault segment^5^. Surface indicators of active tectonic deformation, including depositional and geomorphological features that occur during earthquake events^6–8^, are then used to complement seismological records. Yet, such an approach can be of limited use as geomorphological features that respond to active tectonics are hardly visible at the surface in slowly deforming regions^9^. Sedimentological indicators are only available where recent sedimentation occurs continuously and surface deformation is capable of imposing thickness changes in subsurface strata^10,11^. In addition, local tectonic deformation is best documented in well-layered deposits that settle in calm-water environments (to drape gentle folds and faults) or as mass-transport deposits (landslides), which are usually only triggered by earthquakes with estimated magnitudes of 6.9 or greater in the Richter scale (R)^12,13^. Geomorphologic analyses of active tectonics in slowly deforming karst landscape are even more challenging and, consequently, are rare in the published literature^14^.

**Fig. 1:**
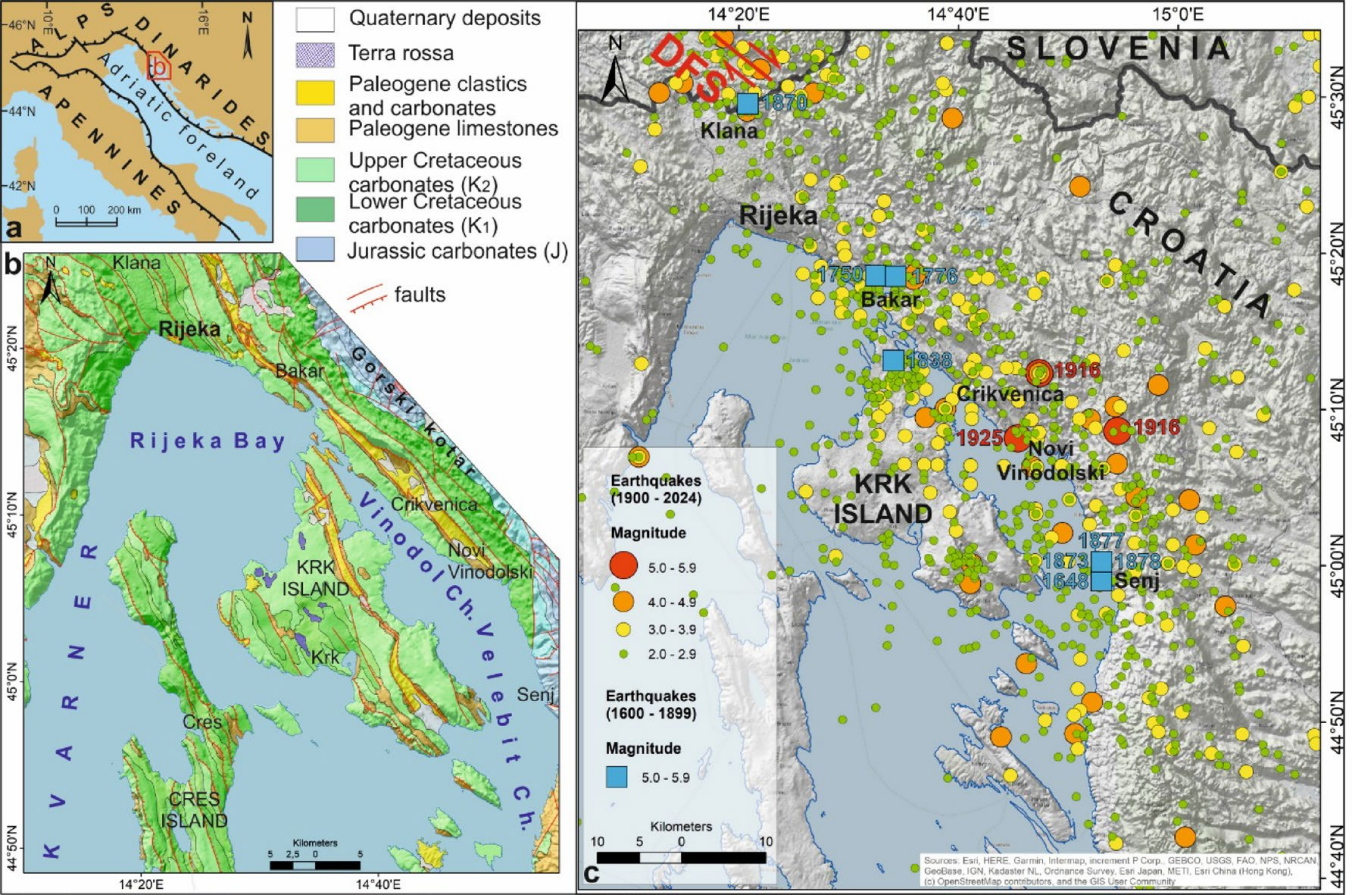
**(a)** Regional setting of the orogenic fronts in the peri-Adriatic region, highlighting the location of the study area in the NW sector of the External Dinarides (Kvarner, Croatia). **(b)** Geological map of the Kvarner area (modified from https://www.hgi-cgs.hr/en/geoloska-karta-republike-hrvatske-1300-000/), showing the location of geographical features of relevance to this work: Rijeka Bay, plus the Vinodol and Velebit channels of the Adriatic Sea. **(c)** Local seismicity gathered from the Croatian Earthquake Catalog (CEC; see Supplementary file 1, and ^17,33^ for more information on seismicity) along the NW-SE striking active fault zone interpreted in this paper, which is named in the neighbouring Slovenia as the Dinaric Fault System (DFS) (see text for references). Numbers in blue indicate years of estimated M > 5R historical earthquakes (locations marked by blue squares), while the numbers in red indicate the year and magnitude of the strongest instrumentally recorded earthquakes (locations marked by red circles), as described in the text.

Active surface deformation is slow along the NW part of the External Dinarides fold-and-thrust belt^15^, with crustal strain distributed along many potentially active faults along the eastern margin of the Adriatic Sea, in Slovenia and Croatia^16–19^ (Fig. 1). Onshore, active faults are recognized in Slovenia, where they are named the Dinaric Fault System (DFS), an up to 50 km wide zone of distributed active strike-slip faulting^16,18^ that likely results from late-orogenic escape tectonics^20^. Such an escape tectonics has been promoted by tectonic transport from the collision zone of the Adriatic microplate (Adria) with Eurasia^20^, which has deformed the broader External Dinarides since the Miocene. Hence, orogen-parallel strike-slip faulting predominates at present in Slovenia and Croatia to partly disrupt: (a) the depositional continuity of foreland successions, and (b) the structural coherence of the thin-skinned Dinaric thrust-and-fold belt^20^.

The Kvarner area, a region of slow tectonic deformation in the External Dinarides, partly extends offshore along the DFS and is characterized by its relatively moderate earthquakes, which may reach a magnitude of 6.7R as estimated from historical data (Supplementary File 1). The latest results of seismological analyses from southern Slovenia, to the NW of the Kvarner area, highlight the existence of active strike-slip faults that are steep (subvertical), dextral in their movement, and penetrate deeply into the crust^21,22^. These faults are active along a zone of thickened crust^22,23^, probably because a regional fault separating two major crustal segments of the Adria, that occurs in southern Slovenia and strikes NW-SE below the main crest of the External Dinarides^24^. There are also published models that interpret dextral strike-slip movements along NW-SE striking faults in the northern part of the Kvarner area and southern Slovenia, based on estimated slip rates for active faults in the External Dinarides^25^.

Despite the above geological models, active strike-slip faulting along the DFS has not yet been documented at the surface along the SE continuation of the DFS towards Croatia^17^. Regional seismological data have revealed subsurface crustal activity in the form of variable fault plane solutions around the Kvarner area^17,21^, but it is challenging to model active faults at the surface because there is no clear correlation between deep seismological data and surface geological data^17^. Compounding this problem, active faults in the Kvarner area are also not too obvious when compared to their counterparts in southern Slovenia, implying that seismogenic sources should be revised for the whole of Croatia (Fig. 1c). Key evidence of possible active deformation would be expected in stratified near-surface deposits, but Quaternary sediments are scarce onshore and on the Kvarner islands. Instead, Quaternary sediments are widespread in the shallow Adriatic Sea around these islands, and also in the multiple bays and inlets that form the broader Kvarner area^26,27^. Crucially, the instrumentally recorded active faults in the NW part of the External Dinarides are not reactivated Dinaric thrusts but crustal transpressional faults that dissect the older (and shallow) Dinaric nappes^21^, and these same nappes may be rearranged into large gravitational and intermittently unstable upper crustal structures^17^. This suggests a greater geohazard risk than previously considered in the Kvarner area and NE Adriatic as a whole, particularly as these are densely populated regions where some of the most important national and central European infrastructure is located.

This work aims to present critical new evidence for active tectonic deformation in the submerged Kvarner area (Fig. 1). New high-resolution sub-bottom shallow-seismic data were acquired for such a purpose, and image the zones that recorded past earthquakes along inferred composite active fault zones^28^. These new data will significantly improve our understanding of the active tectonics recorded in the area, and assist future geohazard assessments.

## Background

### Geological setting

The Kvarner area belongs to the External Dinarides, an intensively deformed pre-orogenic sedimentary cover that was part of the central Adriatic microplate during the Mesozoic^17,24,29^ (Fig. 1). The External Dinarides were mainly deformed in the Eocene and Oligocene as part of a thin-skinned orogenic system^30^. Late-orogenic thick-skinned deformation ensued during the Miocene and resulted in the exhumation of the orogenic system along transpressive corridors^24^. This tectonic mechanism is interpreted to still be active^20^ but, counter-intuitively, there is no reported evidence for active onshore faults over the predominantly karstic terrain of the Kvarner area^17^ (Fig. 1). In summary, the combined influence of multiple factors such as tectonic movement^31–33^, sealevel change, climate and subsurface lithology has resulted in the complex geological and geomorphological settings of the NE part of Kvarner area and its three sub-sectors: Rijeka Bay, the Vinodol Channel and the Velebit Channel^26,34–36^ (Fig. 1b and c). Such a complexity has already been documented offshore by high-resolution seismic reflection surveys in specific Quaternary basins of the wider Kvarner area^27^, but not to its full extent and geodynamic significance.

According to the Croatian Earthquake Catalogue (CEC; Supplementary File 1), the strongest historical earthquakes (> 5R) have occurred in an area spanning from Klana (Rijeka hinterland) to Senj, and were likely related to the DFS (Fig. 1c). The most significant historical earthquakes with epicentres in the study area, recording intensities greater than or equal to VII in the Medvedev–Sponheuer–Karnik scale (°MSK), occurred in 1323, 1648, 1750, 1776, 1838, 1870, 1873, 1877 and 1878, the strongest of which occurred in 1323 near Crikvenica with a maximum intensity of IX °MSK and an estimated magnitude of 6.7R. Alas, historical data about this earthquake are unreliable^17,33^ and this event is not included on the map in Fig. 1c. After this early 14th -century earthquake there were no more significant events until 1648, when an earthquake with an epicentre north of Senj occurred with an estimated magnitude of 5.3R. In 1750, a series of earthquakes with a maximum intensity of VII-VIII °MSK occurred near the town of Bakar (Fig. 1c). Their maximum estimated magnitude was 5.6R. Later, a relatively strong earthquake occurred in 1776 near the town of Bakar, also affecting the town of Rijeka. It had an assigned maximum intensity of VII °MSK and an estimated magnitude of 5.3R. The strongest earthquake on the island of Krk occurred in 1838. Maximum intensity was VII °MSK (for an estimated magnitude of 5.2R), and it was particularly felt on the northern part of the island. In 1870, there were four earthquakes in the area of Klana with magnitudes greater than 4.5R. This earthquake sequence included a main earthquake with a maximum intensity of VIII °MSK (estimated magnitude of 5.5R) followed by multiple aftershocks, the strongest of which reached magnitudes of 4.6R, 4.7R, and 4.9R, all occurring within three months of the main event. The main earthquake had an epicentre just north-northwest of Klana, while the three subsequent (and large) aftershocks were located southwest to south-southeast of Klana. The epicentres of earthquakes near Senj occurred in 1873, 1877 and 1878, in the same location, with all three earthquakes recording the same maximum intensity VII °MSK and an estimated magnitude of 5.2R.

The strongest instrumentally recorded earthquakes with magnitudes greater than or equal to 5 were in 1916 (two events) and 1925, with magnitudes of 5.8, 5.4, and 5.2, respectively. The epicentres of these earthquakes were located in Novi Vinodolski itself and its surroundings (Fig. 1c). Recorded magnitudes of these events agree with the estimated maximum magnitudes of historical events in the Kvarner area (CEC; Supplementary File 1).

## Results

### High-resolution seismic profiles

**Fig. 2:**
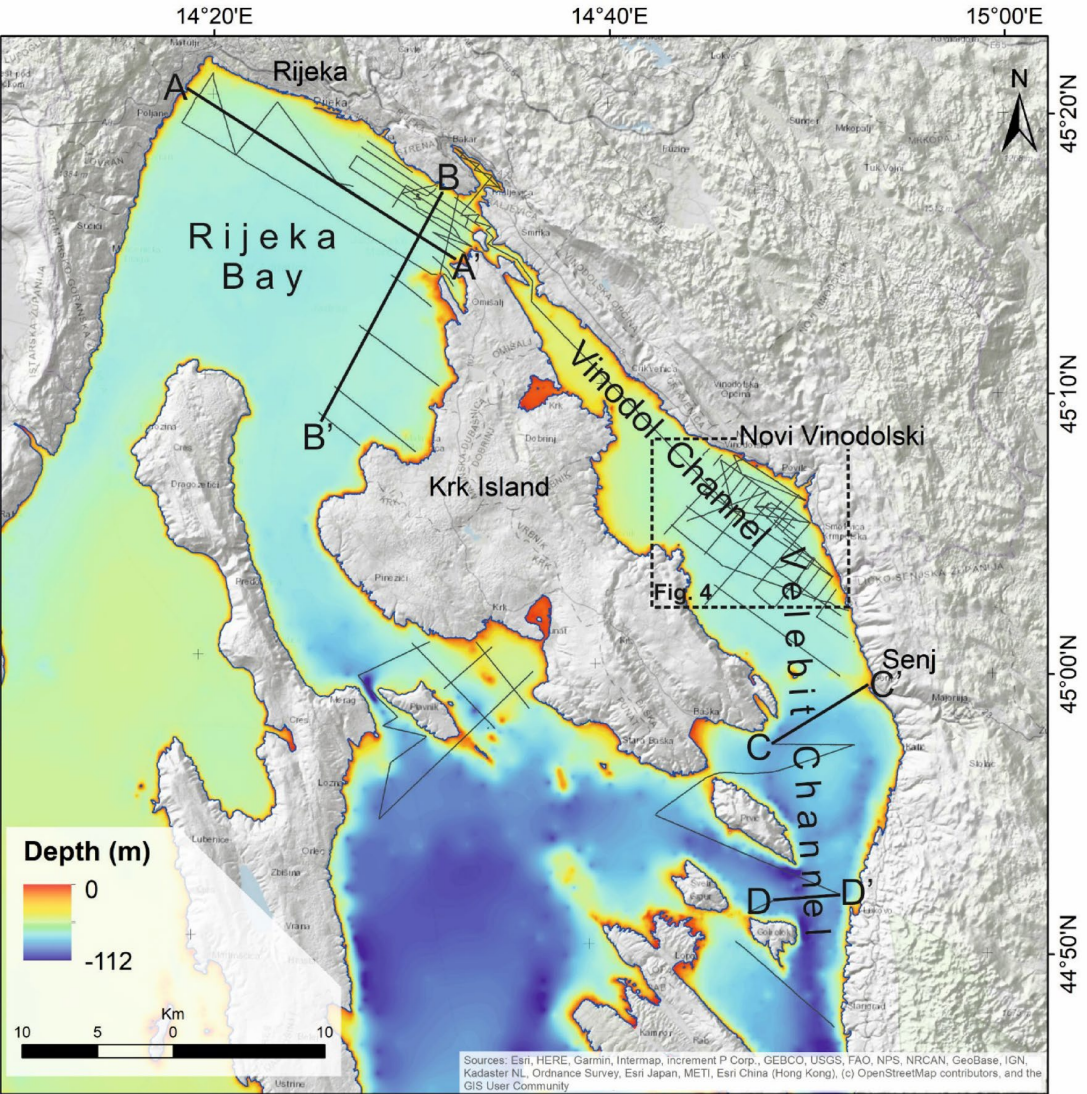
Location map of the high-resolution seismic survey in the Kvarner area (black tracklines). Selected profiles A-A’, B-B’, C-C’ and D-D’ are interpreted in Fig. 3.

**Fig. 3:**
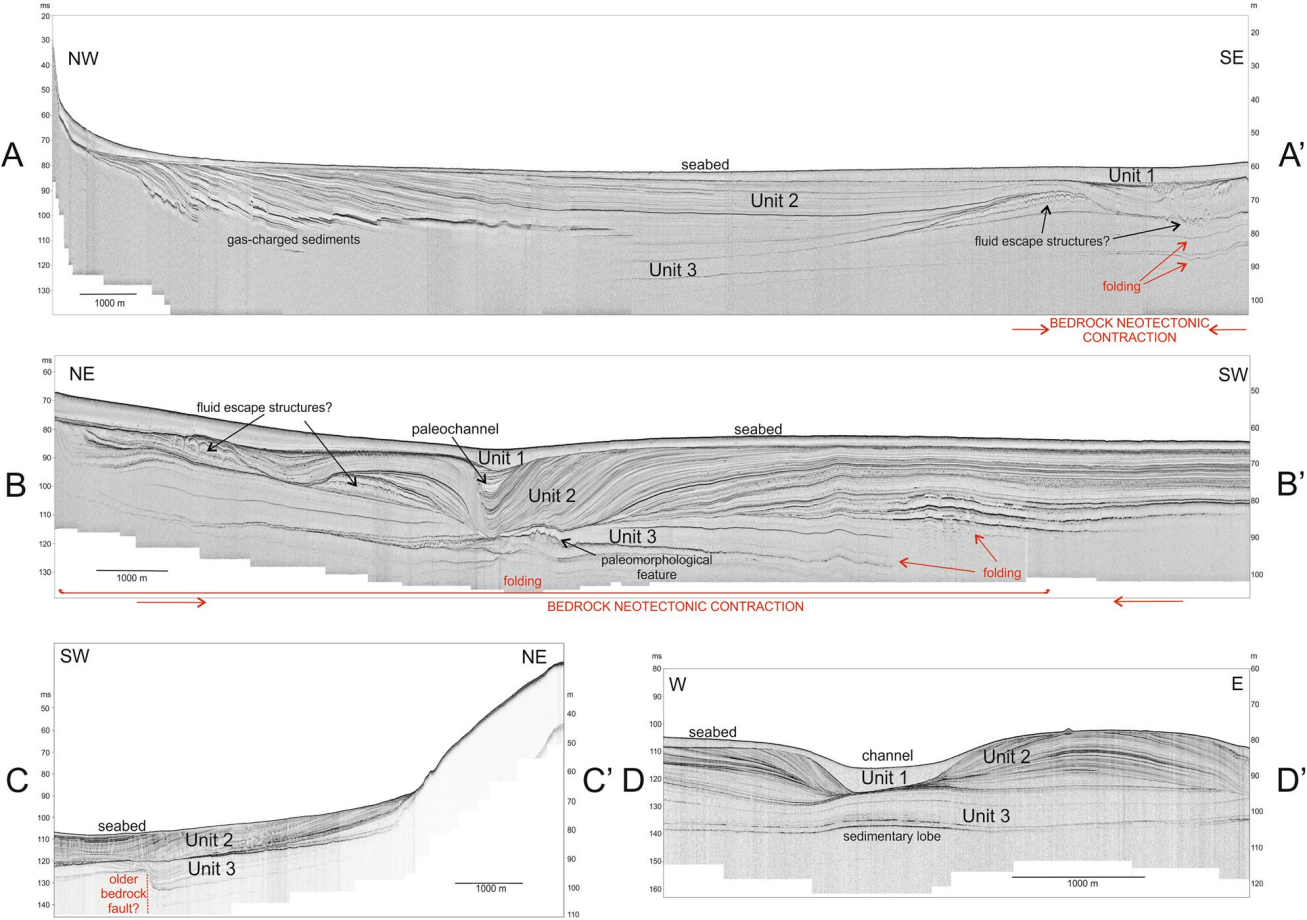
Selected high-resolution seismic profiles from the NE Kvarner area showing the styles of neotectonic deformation affecting Quaternary sediments. Active faults are not observed near the seafloor. Note the tectonic folding and faulting of the older Quaternary strata in Unit 3 (profiles A-A’, B-B’) in response to the neotectonic movements. The location of the profiles and regional bathymetric data is given in Fig. 2.

**Fig. 4:**
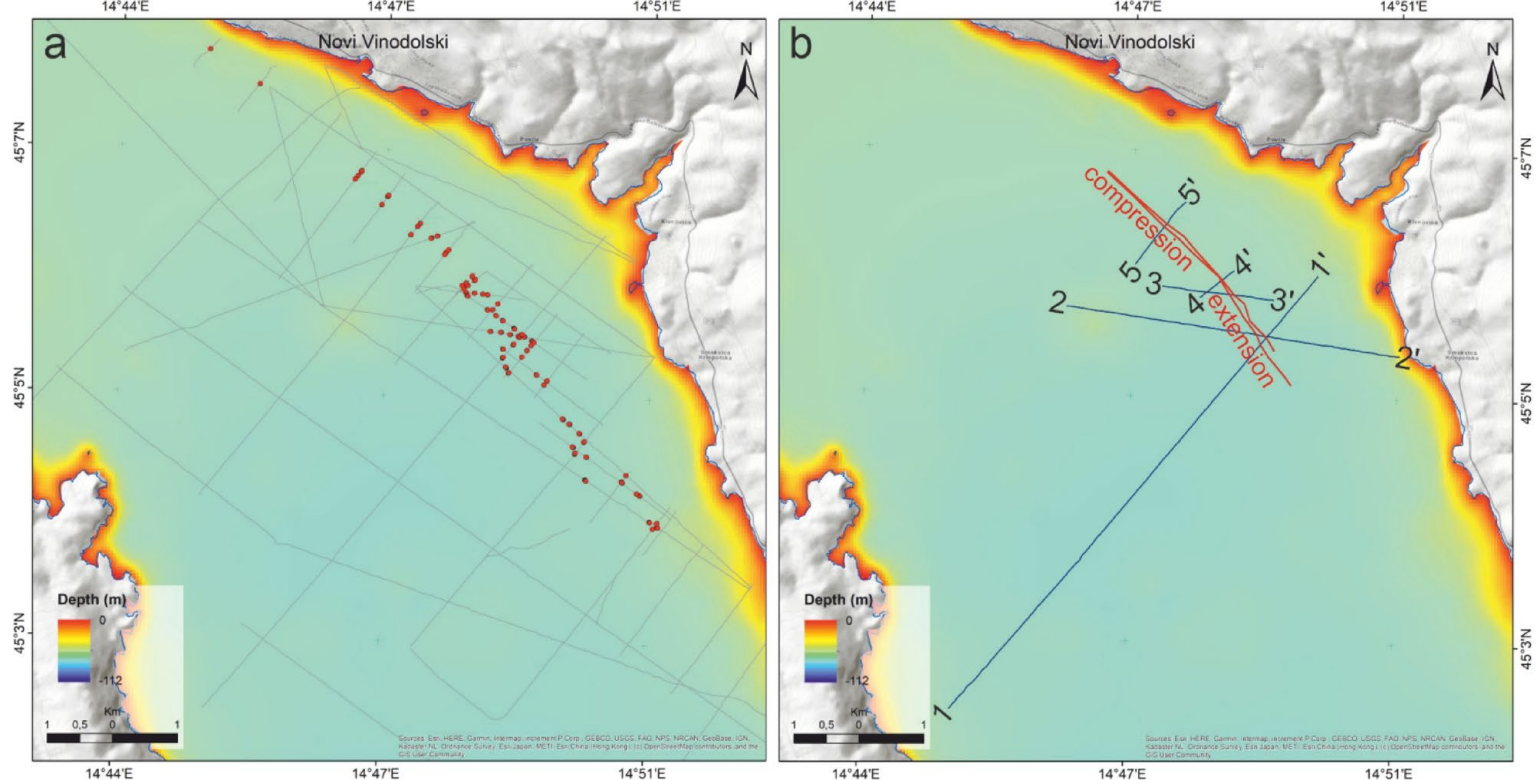
**(a)** Tracklines of all high-resolution seismic profiles acquired near Novi Vinodolski with red dots indicating intersection points with the neotectonic faults observed on the profiles. **(b)** Active faults (red lines) from the neotectonic fault zone and the tracklines of selected profiles are shown in Fig. 5.

**Fig. 5:**
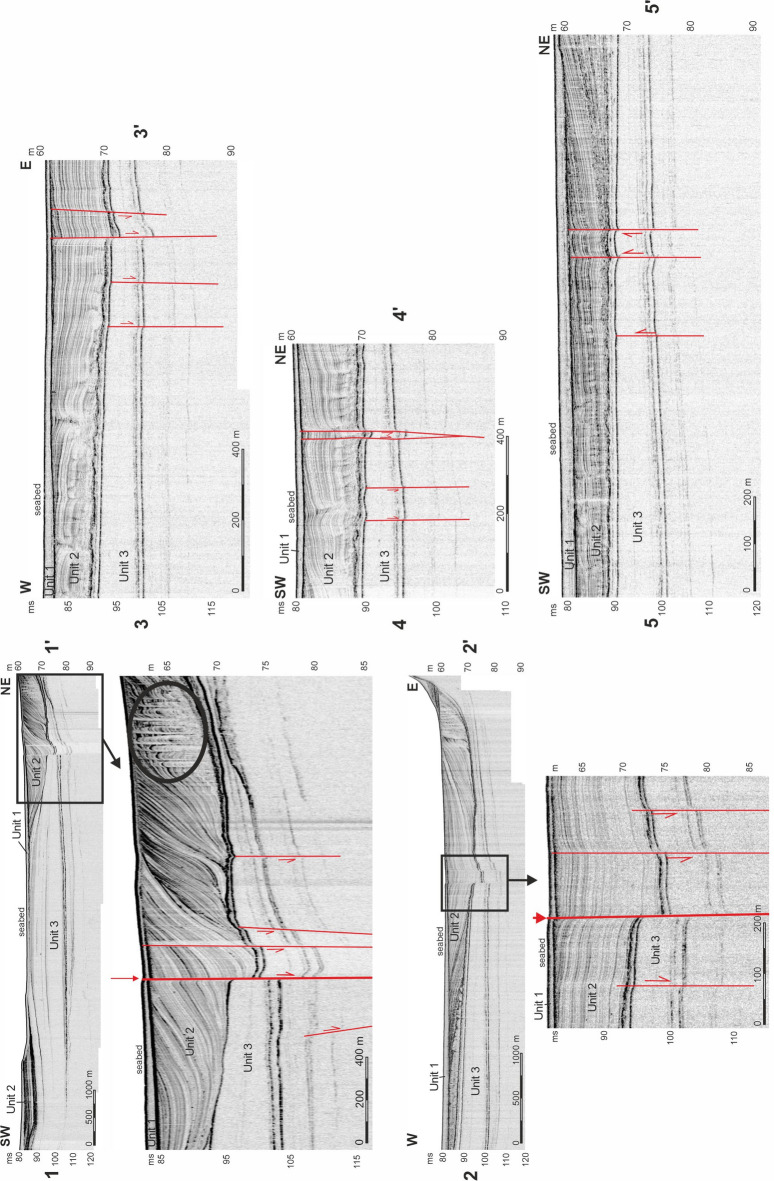
Selected high-resolution seismic profiles across the active subvertical fault zone near the Vinodol-Velebit channels (see their location in Fig. 4). The red arrows in profiles 1–1’ and 2–2’ highlight a seabed scarp related to active faulting. Note the predominantly normal fault offsets and the small graben structures on profiles 1–1’, 2–2’, 3–3’, 4–4’, all denoting local extension. A positive structure denoting local compression is recognized on profile 5–5’.

Three near-seafloor (Quaternary) sediment packages, or units, were identified in new high-resolution sub-bottom seismic images as shown in Figs. 2, 3, 4 and 5. Two of these units are nearly transparent and contain only a few internal seismic reflections (Units 1 and 3). In between occurs a unit with sub-parallel, divergent and contorted internal reflections (Unit 2). We attribute the topmost package (Unit 1) to the Holocene, whereas below are inferred late Pleistocene sediments (Units 2 and 3). These ages were estimated based on correlations with regional seismic records and radiocarbon-dated samples from sediment cores^27^.

### Rijeka bay and South Velebit channel

The imaged Quaternary strata reveal multiple sedimentary sequences that are bounded by marked erosional surfaces (Figs. 3 and 5). Seabed channel erosion is interpreted south of the island of Krk (Fig. 3), but high-resolution seismic profiles from the Rijeka Bay and the southern part of Velebit Channel do not reveal active surface faulting in this area, nor do they show fault offsets at the base of Unit 1 (Fig. 3). However, there is gentle folding in Unit 3 in the eastern part of the Rijeka Bay (Fig. 3, profile B-B’). Neotectonic compression of the bedrock should be responsible for the gentle folding of superficial strata observed in profile B-B’, which affected the older part of the well-bedded Unit 2 (Fig. 3).

High-resolution seismic profiles imaging the Velebit Channel between Krk and Senj (Fig. 2) denote important offsets in Unit 3 that are probably related to neotectonic movements that are older than Unit 3 (Fig. 3, profile C-C’). An elongated bathymetric low (channel), suggestive of active subsidence in this sector, is also identified; it strikes NNW-SSE along the southern part of the Velebit Channel (Fig. 2). However, the selected profile D-D’ in Fig. 3 shows that this trough is not related to neotectonic deformation as there is no evidence of active faulting (or folding) in Quaternary strata.

### Vinodol and North Velebit channels

High-resolution seismic profiles acquired in the Vinodol and the northern part of the Velebit channels (Fig. 4) reveal active deformation in Quaternary deposits. In particular, a > 100 meter-wide fault zone, limited by parallel subvertical fault planes, is identified in seismic data (Fig. 5). Small normal faults are observed along the SE part of this fault zone, with kinematic/structural indicators suggesting important NE-SW extension (Fig. 5, profiles 1–1’, 2–2’, 3–3’, 4–4’). Conversely, in the NW part of the mapped zone, seismic profiles show strata uplift between two parallel faults, a character suggesting NE-SW compression (Fig. 5, profile 5–5’).

The main active fault zone in the Vinodol and North Velebit Channel records a vertical displacement of up to one meter during the Holocene, and a few meters in the late Pleistocene (Fig. 5, profiles 1–1’ and 2–2’). This fault is identified on the seafloor as a small seabed scarp (Fig. 5, profiles 1–1’ and 2–2’). There are inactive neotectonic faults that strike parallel to the active faults imaged at a distance of up to 200 m to the east and west of the main fault zone. Importantly, these inactive faults do not offset upper Pleistocene strata in Unit 2 (Fig. 5). Finally, convolute intervals are observed in Unit 2 as hyperbolic reflections that contrast with the well-stratified intervals identified in other parts of the seismic profile (Fig. 5, profile 1–1’).

### Fault mechanism solutions

**Fig. 6:**
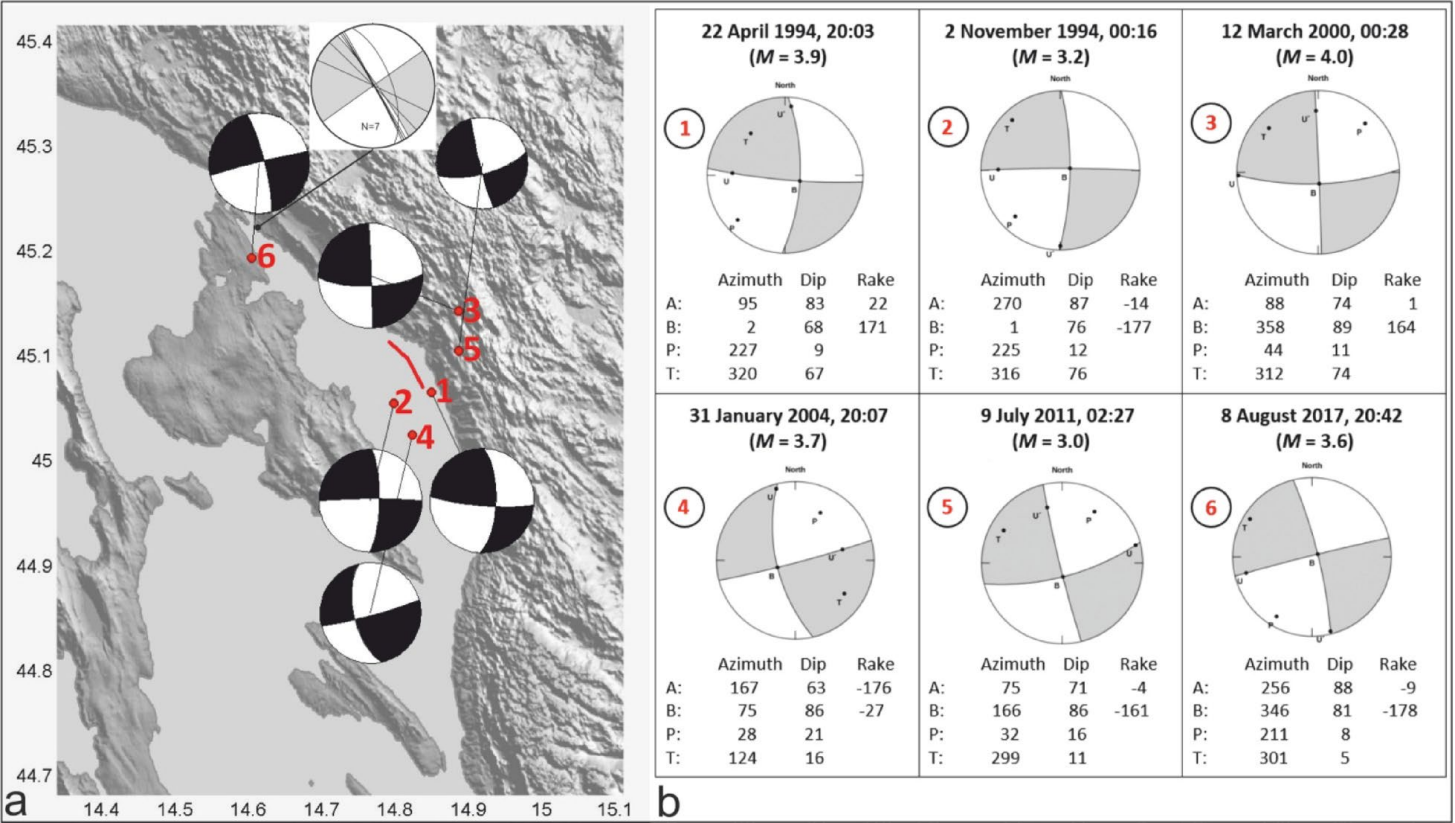
**(a)** Map of the selected earthquakes recorded in the research area, and their Fault-Mechanism Solutions (FMSs, black-and-white “beachballs”, details in “b”). Inset grey-and-white beachball showing seven measured dextral strike-slip fault planes on the field (45° 13′ 26.4″ N, 14° 36′ 38.5″ N). The red line highlights the location of the active fault zone mapped in this work using high-resolution seismic data. **(b)** Calculated FMSs for selected moderate earthquakes according to the Croatian Earthquake Catalogue (CEC, the locations of the seismic stations are given in^43^). A stereographic projection of the lower focal hemisphere was used. Azimuth, dip, and rake mark the azimuth of the fault strike, fault dip and the direction of movement along the fault surface. The projections of the directions of maximum compression and dilation are referred to as the P- and T-axes, respectively. U corresponds to the projection of the slip vector of the hanging wall of the fault in relation to the footwall. U´ corresponds to the direction of the slip on the auxiliary plane, which would result in the same spatial distribution of the radiated seismic energy if the slip had occurred on the auxiliary plane. The B-axis corresponds to the intersection between the fault plane and the auxiliary plane.

**Fig. 7:**
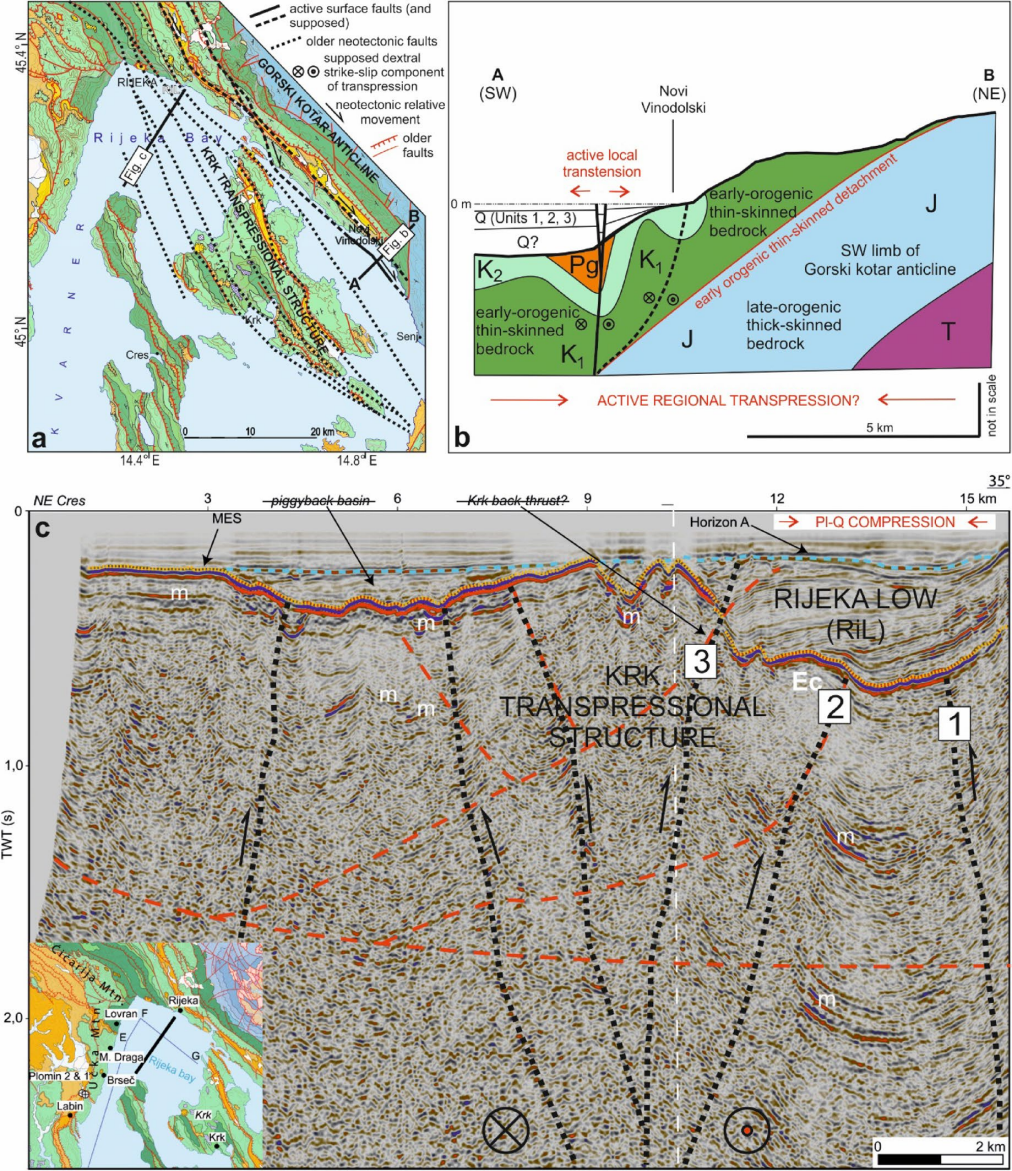
**(a)** Overview map of interpreted neotectonic (Plio-Quaternary) faults in the Kvarner area (black lines), part of the Dinaric Fault System (DFS). See the geological map in Fig. 1b for the legend. **(b)** Schematic cross-section A-B summarizing the mechanisms that promote active surface faulting in the Novi Vinodolski area, near the coast (see Fig. 6a for location). Q – Quaternary superficial deposits (Units 1, 2 and 3 imaged in high-resolution seismic data). T – Triassic, J – Jurassic, K_1_ – Lower Cretaceous, K_2_ – Upper Cretaceous, and Pg – Paleogene bedrock units. **(c)** Reinterpretation of a deep-seismic profile across the Rijeka Bay taken from^31^. Interpreted neotectonic faults are marked as black dotted lines. Numbers 1, 2 and 3 refer to the relative timing of activation of the faults in relation to the deposition within the ‘Rijeka Low’, a localized Pliocene basin formed in this area above the Messinian unconformity (MES). m – multiple, Ec – interpreted Eocene flysch. The strikethrough text in the figure highlights the reinterpreted nature of basins and major structures in this work.

Fault mechanism solutions (FMSs) offer insights into the geometry of earthquake sources, e.g., they provide crucial data for the characterization of active faults, enabling research in tectonics and structural geology. They are computed using the spatial distribution of the observed P-wave first motion polarities of earthquake records from a number of well azimuthally distributed stations. FMSs in the wider Kvarner area indicate the presence of strike-slip faults that trend predominantly NW and NE^43^. In the Vinodol-Velebit channel area, recalculated FMSs suggest the presence of strike-slip faults with differing NNW-SSE, N-S, W-E, and WSW-ENE strikes (Fig. 6 and Supplementary File 1). Significantly, these strikes are neither parallel to the trends of faults identified in the sub-bottom profiler data, nor to active surface faults inferred onshore (Fig. 7). However, rare fault planes measured in the field along the active fault zone indicate the presence of NW-trending strike-slip faults (Fig. 6a) that, in combination with the nearby FMSs, imply a predominant dextral movement near the surface.

## Discussion

### Active tectonics along offshore fault zones

**Fig. 8:**
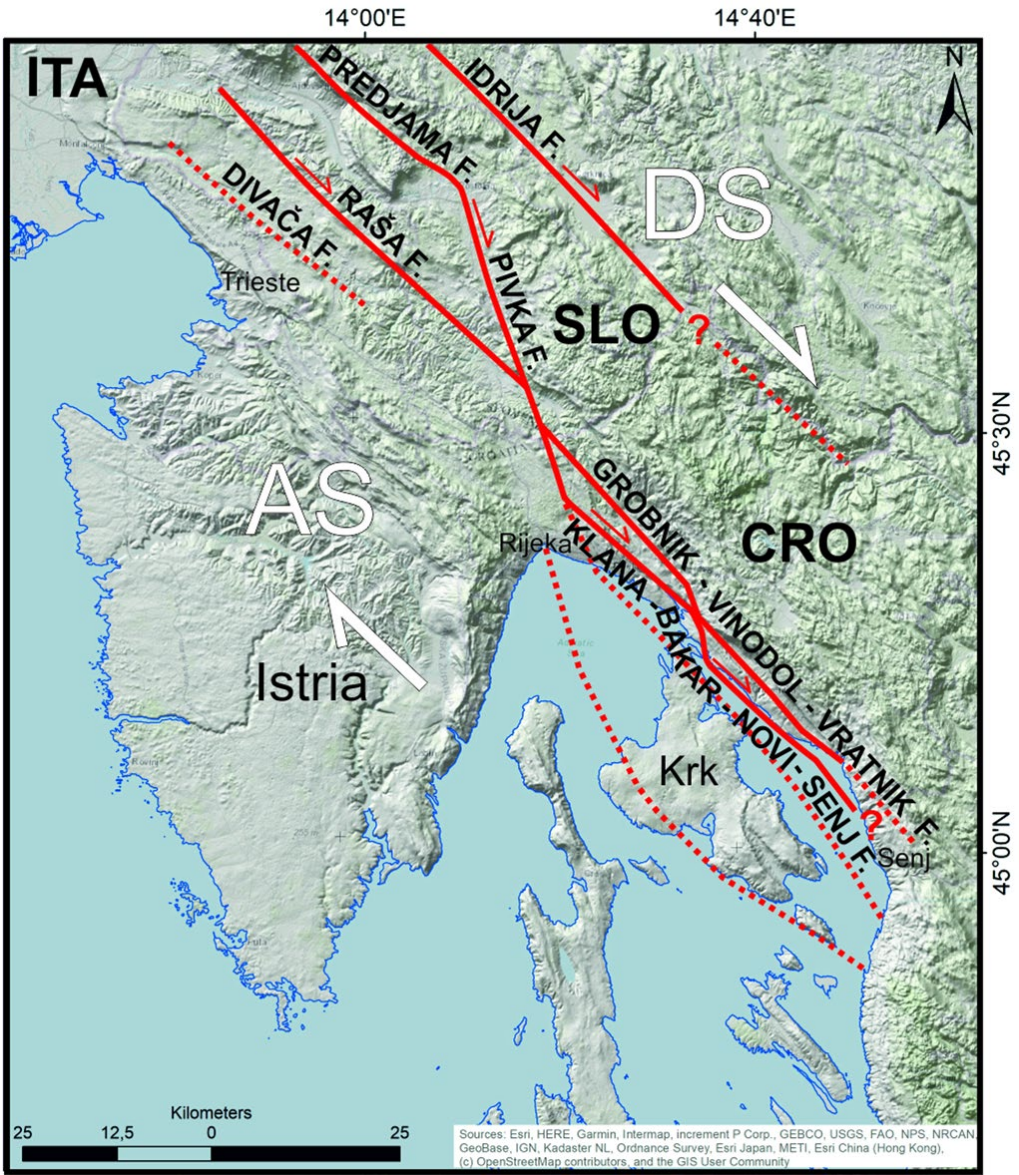
Overview schematic map of presumably active (thick red lines) and potentially active (dotted red lines) neotectonic surface faults in the Dinaric Fault System (DFS), NW External Dinarides, according to^17,18,21,22,28^ and this work. DS – Dinaric segment, AS – Adriatic segment of the Adria lower crust^24^.

The wide zone of gentle folding in the older Quaternary strata, identified towards the NE part of the Rijeka Bay (Fig. 3, profile B-B’), is considered to reflect neotectonic shortening of the bedrock. The faults offsetting the bedrock in this zone are tentatively correlated with highly fractured carbonates on land, which are known for their enhanced weathering^16^. In comparison, steep neotectonic reverse faults offsetting older bedrock units in the coastal area of Rijeka and Novi Vinodolski, as well as in Vinodol and Velebit channels (Fig. 7a, b), are probably part of the active strike-slip DFS mapped in southern Slovenia^16,18^. These seismogenic faults in southern Slovenia are seismologically modeled as steep and penetrating deep into the crust^21,22^, while one of the most recently active structure strikes SE towards Croatia and is named here as the Pivka fault (Fig. 8).

Active faults along the DFS of the Kvarner area are interpreted in this work as accommodating the ongoing transpression of reactivated thick-skinned reverse faults that control the exhumation of the External Dinarides^24^. Tectonic strain is probably dissipated near the surface along reactivated and rearranged thin-skinned structures, e.g., basal décollements and axial-plane structures associated with kilometer-scale folds^17^ (Fig. 7b). It has been reported from multiple fold-and-thrust belts that reactivated reverse faults accommodate transpressional movements in the crust^37,38^. A similar mechanism is thought to be responsible for the late-orogenic exhumation of the External Dinarides^20,24^, as well as for the active tectonics that generates modern earthquakes along this same orogen^39,40^. In such a setting, active transpressional faults are considered to be reactivated pre-orogenic normal faults that separate the Dinaric and Adriatic segments of the Adria crust^24,40^.

The available deep-seismic data recorded in the Kvarner region are of relatively low quality below the Messinian unconformity; yet, the data have been used previously for a tentative interpretation of deep offshore structures^31^. Notably, the bedrock structure in the neighbouring coastal area^16^ does not concur with previous structural interpretations, and a key published seismic profile is reinterpreted in this paper (Fig. 7c). A first important aspect in our reinterpretation is that only Pliocene-Quaternary successions and structures are well-imaged on this profile and, as a result, a post-Messinian basin previously identified in the Rijeka Bay and named Rijeka Low (RiL)^31^, is considered here as separating two neotectonic thick-skinned transpressional structures, the Gorski Kotar anticline and the Krk positive flower structure (Fig. 7). Hence, a first key outcome of our analysis is that it considers this positive flower structure to be a better structural solution for the Kvarner area than the Krk backthrust suggested in previous work^31^ (Fig. 7). The RiL basin was probably developed during the early Pliocene in front of the Gorski Kotar transpressional structure and later deformed in its southern part because of: (a) late Pliocene activity of faults along the island of Krk, and (b) the subsequent development of the Krk transpressional structure (Fig. 7).

The gentle folding of upper Pleistocene strata in profiles A-A’ and B-B’, acquired along the NE coasts of the Rijeka Bay (Fig. 5), indicates neotectonic compression in front of the huge Gorski Kotar transpressional structure, which could still be active. In addition, the relatively low recent seismicity recorded in the study area (Fig. 1c), and the gentle folding of its late Pleistocene strata, suggest very moderate compression in the bedrock. Hyperbolic reflections (convoluted beds) observed locally within Unit 2 show intra-formational soft-sediment deformation (liquefaction) that is likely related to the late Pleistocene earthquakes^17^. There are no folds or faults visible within the youngest Quaternary depositional units of the Rijeka Bay (Fig. 3, profiles A-A’ and B-B’).

Contrasting with the Rijeka Bay, clear evidence for active fault offsets is unequivocal in the Vinodol and northern part of Velebit channels (Fig. 5). Here, our high-resolution seismic data reveal an active NW-SE striking subvertical fault zone (Figs. 5 and 6). Some of the seismic profiles show normal offsets near the seafloor, indicative of local extension, while other profiles reveal local uplift and compression. Compressional features are observed along the NW part of the main NW-SE striking fault zone, while tectonic extension is observed along its SE part (Fig. 5). The geometry of the fault zone, especially the presence of negative and positive structures that alternate along its strike, is typical for strike-slip faults^41^. In other words, the switch from compression to extension is a likely consequence of small changes in the strike of faults within a larger strike-slip fault zone, forming local restraining and releasing bends^42^.

The displacement of subvertical faults in the Vinodol-Velebit channel increases with depth, implying that faults in this region have been active during at least the late Pleistocene (Fig. 5). The well-stratified Unit 2 has been interpreted as deposited during the latest terrestrial phase affecting the area, i.e., during the last glaciation, which lasted for approximately 100,000 years^27^. Thus, the few meters of vertical displacement observed at the base of Unit 2 can be correlated with that period. In contrast to the upper Pleistocene Unit 2, surface rupture (seabed scarp) is only observed in a small section of the NW-SE striking fault zone (Fig. 5, profiles 1–1’, 2–2’). Strikingly, horizontal displacement along the NW-SE striking fault zone is not apparent on the acquired seismic profiles.

Similar to the Rijeka Bay, intra-formational hyperbolic structures are observed locally along neotectonic fault zones in the well-stratified Unit 2, and these suggest local sediment liquefaction. They resemble fluid/sediment escape structures that relate to strong shaking during prehistorical (Pleistocene) earthquakes, as recognized near similar structures in the wider Adriatic area^8^ (Fig. 5, profile 1–1’).

### Geodynamic implications

Seismological data, i.e., the fault-mechanism solutions (FMSs) of modern earthquakes shown in Fig. 6, prove that the Kvarner area is intersected by deep-rooted strike-slip faults, some of which are tentatively interpreted in low-quality deep-seismic data (Fig. 7). This observation agrees with studies demonstrating strike- and oblique-slip fault mechanisms in the NW and SE continuation of a neotectonic fault zone present in the region^17,24,43^. Yet, there is no strict overlap between the strike or locations of recognized earthquake epicenters and the active fault zone mapped in this work (Figs. 6 and 7a). This causes problems when characterizing seismogenic hazard in one of the most populated regions of the Adriatic Sea. The FMSs in Fig. 6 also show that active seismogenic structures are likely dextral strike-slip faults. However, our surface geological mapping reveals only a few dextral strike-slip faults with the general NW-SE Dinaric strike along the NW continuation of the mapped active fault zone (inset in Fig. 6a). Based on this fact, we interpret that the seismologically active faults in the Vinodol-Velebit channels are mostly dextral in their movement (Fig. 7). As the strike of the mapped NW-SE fault zone is not the same as the surrounding seismologically calculated FMSs, crustal strain is interpreted to be redistributed within the deforming thin-skinned overburden strata of the Kvarner area (Fig. 7b). Hence, dextral plane solutions are more probable but we cannot exclude also possible co-seismic slip along minor, secondary left-lateral faults.

The observed discrepancy between the position of faults in seismic data and the epicentre locations of measured earthquakes (Fig. 6a) may result from aseismic slip along the fault close to the surface or, instead, from the expected inaccuracy of epicenter locations, which can reach a few hundred meters when calculated by multiple seismological stations^44^. Active faults in the NE Kvarner area may therefore record both seismic and aseismic slip along the DFS^9^. Regarding the existence of aseismic slip, one should stress that because of the complex geological setting of the Kvarner area, tectonic strain is redistributed from seismologically recognized active faults deeper in the crust to some of the mapped preexisting faults near the surface, and also to newly formed faults along weak zones (e.g., synclines) in the thin-skinned cover^17^. Strain could have also been redistributed near the surface, from one near-surface fault to another, during the late Quaternary^17^. This phenomenon is documented across the neotectonic fault zone in the Vinodol-Velebit Channel, which comprises active and inactive fault segments that occur together with blind neotectonic faults formed laterally and parallel to the main active fault zone (Figs. 4 and 5).

At a regional scale of analysis, the NW-SE striking DFS is probably active further to the SE of the Kvarner area, but some of the active faults could be blind, i.e., active below the thin-skinned overburden, as reviewed by^40^. If this is the case in the Kvarner area, some of the recorded earthquakes could be related to blind faults, since the surface active faults are proven only in the area of the eastern Vinodol and the northern Velebit channels, while there is no surface faulting visible within the deposits that overlie a supposed neotectonic Krk transpressional structure, i.e., NW and SE of the island of Krk (Figs. 3 and 7a and c). However, tectonic strain along major seismogenic thick-skinned faults is likely redistributed during and after major earthquakes within the thin-skinned bedrock deformed during the first phase of the Dinaride orogeny^24^ (Fig. 7b).

Based on the analysis of the hypocenters of the instrumentally recorded weak earthquakes, active segments of seismologically modeled transverse faults in the area of Bakar offset the crust at significant depths^17^, with these segments being generally perpendicular to the DFS, which strikes NW-SE. However, analyses of deep geological structures in the western Kvarner area^31^, and its southern offshore sector^32^, indicate that transverse N-S to NE-SW striking faults were active in that part of the Kvarner area only until the late Miocene. Nevertheless, possible creeping activity of northeastern portions of the supposed deep crustal active transverse fault network^24^ could be responsible for the near surface formation of local restraining and releasing bends that could be responsible for the above mentioned shift from contraction to extension at the transition between the Vinodol and Velebit channels (Fig. 4), as well as for the sigmoidal configuration of the DFS networks (Fig. 8).

As a corollary of this work, we interpret that the surface neotectonic faults that follow a NW-SE strike in the Kvarner area, and belong to the DFS, were controlled by deeper structures that are active below the thin-skinned overburden succession deformed during the earlier phase of the Dinaride orogeny. Yet, the strikes of recognized shallow and deeper active faults are not exactly parallel, denoting complex reactivation styles. The mapped faults could nevertheless take over the strain redistributed deeper in the crust, scattering it near the surface along sigmoidal (braided) active fault segments (Figs. 7a and 8).

As the complex active tectonic system in the Kvarner area is still not completely defined, long-term inter-seismic monitoring and occasional co-seismic observations of surface deformation are paramount to understand the degree of activity of the DFS. Fault-related movement should be properly documented with an established net of GPS points, while a deeper seismic imaging of Cenozoic sedimentary cover in the wider Kvarner area will certainly result in the recognition of new neotectonic structures. In parallel, analyses of the new LIDAR coverage of Croatia’s onshore area will lead to the acquisition of new geomorphological evidence for active faulting.

## Summary and conclusions

An integrated approach to identifying active seismogenic faults in a key region of the Dinaric Fault System was attained in this work. The analyses provide novel insights into the neotectonic evolution of the Kvarner area as follows:

- Gently folded surface (Quaternary) depositional units in the NE part of Rijeka Bay document an active but slow transpression of the bedrock along the Dinaric Fault System (DFS), with this transpression being responsible for the instrumentally recorded earthquakes in that part of the External Dinarides.
- The generally NW-SE striking subvertical active fault zone detected in near-seabed deposits in the Vinodol Channel and the northern part of the Velebit Channel has the hallmark characteristics of a strike-slip fault zone.
- A switch from tectonic contraction along the NW part of the recognized segment of the active fault zone in the Vinodol-Velebit channel, to tectonic extension along its SE part, may be a consequence of variations in the direction of the NW-SE fault zone and the presence of deeper structural elements that can modify its character near the surface.
- Discrepancies between the strike of the active deeper crustal faults, calculated from the seismological data and the strike of the mapped active fault near the seafloor, result from the above-mentioned redistribution of the strain near the surface, along the NW-SE-trending, strike-slip faults belonging to the active DFS.

This work provides new evidence for active surface faulting in the Kvarner area. This is a particularly significant result considering that the area is one of the most densely populated regions in Croatia and hosts some of the economically most important national and central European infrastructure. Further research should be focused on the active fault zone recognised in this work and its presumed onshore continuation, as future surface earthquake rupture may damage buildings and other infrastructure. Ultimately, the results in this work have an impact on future geohazard assessments along the DFS.

## Materials and methods

 To detect possible submarine active deformation, we acquired new high-resolution seismic-reflection data using an Innomar SES-2000 sub-bottom profiler (Figs. 2, 3, 4 and 5). The Innomar SES-2000 is one of the Innomar parametric sub-bottom profilers (SBP, also called "sediment echo sounder") used to image sub-seabed structures and stratigraphy or objects within the seafloor or river beds. It provides unequalled high-resolution sub-bottom data, and was chosen as the most suitable as it is designed for application at water depths down to 400 m. Acoustic penetration of this system can reach 50 m^45,46^ with a vertical resolution of 5 to 10 cm^47,48^, which was optimal for the purposes of this study. The device uses a high frequency for bottom tracking and a low frequency for sub-bottom data. In conjunction with a high ping rate, it provides good sound-wave penetration and a high resolution of subsurface data. The Innomar system was previously used in similar environments to the Kvarner area as documented in^49–52^. 

 The sub-bottom profiler survey was set at a low frequency of 6, 8 kHz and 12 kHz. The transducer was side-mounted on a 6-m-long vessel. We used Applanix POS MV WaveMaster with two Trimble GNSS antennas and an RTK unit to receive navigational data from the CROPOS (Croatian Positioning System) network. We also adopted a slow vessel speed of 3.5 knots during seismic acquisition. The digitally recorded data was converted to SEGY format, then processed and interpreted in GeoSuite Allworks^®^. The acoustic signal penetrated up to a depth of up to 39 m into the sediment, whereas a velocity of 1514 m/s was used for time-to depth conversion^53,54^. We recorded 65 acoustic profiles with a total length of 264 km (Fig.2). Final structural maps were created in ArcGIS 10.2^®^.

 Seismological records for the Kvarner area were obtained from the Croatian Earthquake Catalog (CEC, Supplementary file 1) and its updated version first described in^19^. For this paper, fault-plane mechanisms were recalculated on the basis of all available data on the P-wave first motion of earthquakes with magnitudes greater than or equal to 3 recorded in the study area. Although moment tensor inversion techniques (MT) tend to prevail in the literature, especially for large events, the FMS method still provides valuable information. The MT is today restricted mostly to earthquakes of magnitudes larger than approximately 3.6–5.5, so smaller ones are commonly analysed by FMS algorithms. As the earthquakes analyzed in this work were of small magnitudes, it was decided to calculate FMSs.

 Since the seismological calculations in this research are primarily focused on weaker instrumentally recorded modern earthquakes (1994 onward), with magnitudes up to 4.5R, while the seismogenic fault characteristics (length, slip, displacement) are uncertain or poorly constrained, the input variables for empirical scaling relationships (as proposed by^55^) are highly unreliable. For this reason, it was concluded that relationships for magnitude estimation lack scientific validity due to poor data reliability, extrapolation beyond the calibration range, and the breakdown of scaling behaviour at small magnitudes. Therefore, they are not applied in this paper.

## Supplementary Information

Below is the link to the electronic supplementary material.

Supplementary Material 1

Supplementary Material 2

Supplementary Material 3

Supplementary Material 4

Supplementary Material 5

## Data Availability

Complete seismological data from the Croatian Earthquake Catalogue (CEC) are available for scientific purpose upon written request to the Department of Geophysics, Faculty of Science, University of Zagreb; however, we provide selected seismological data in Supplementary File 1. High-resolution seismic data are available for scientific purpose upon written request to the Croatian Geological Survey. We nevertheless provide uninterpreted seismic profile images presented in Supplementary Files 2, 3, 4.

## References

[1] Mukherjee, S., Dasgupta, S. & Alsop, G. I. Structural geology in active tectonic regions: an introduction. *J. Str. Geol.* **198**, 105443 (2025).

[2] De la Peña, L. G. et al. Yelles-Chaouche, A. Evidence for a developing plate boundary in the Western mediterranean. *Nat. Comm.* **13**, 4786 (2022). 10.1038/s41467-022-31895-z PMC9378692 35970846

[3] Nishuyama, N. et al. Analysis of the stress field around concealed active fault from minor faults-slip data collected by Geological Survey: An example in the 1984 Western Nagano earthquake region. Earth and Space Sci. **11**, eEA003360 (2024). (2023).

[4] Wu, Z. & Hu, M. Neotectonics, active tectonics and earthquake geology: terminology, applications and advances. *J. Geodyn.* **127**, 1–15 (2019).

[5] Ellis, S. et al. New Zealand fault-rupture depth model v.1.0: A provisional estimate of the maximum depth of seismic rupture on new zealand’s active faults. *GSA Bull.* **114**, 78–94 (2023).

[6] Liu, C. et al. Complex multi-fault rupture and triggering during the 2023 earthquake doublet in southeastern Türkiye. *Nat. Comm.* **14**, 5564 (2023). 10.1038/s41467-023-41404-5 PMC10492857 37689816

[7] Ren, C. et al. Supershear triggering and cascading fault ruptures of the 2023 Kahramanmaraş, Türkiye, earthquake doublet. *Science* **398**, 305–311 (2024). 10.1126/science.adi1519 38236973

[8] Trincardi, F., Cattaneo, A., Corregiari, A. & Ridente, D. Evidence of soft sediment deformation, fluid escape, sediment failure and regional weak layers within the late quaternary mud deposits of the Adriatic sea. *Mar. Geo*. **213**, 91–119 (2004).

[9] Landgraf, A., Kubler, S., Hintersberger, E. & Stein, S. Seismicity, fault rupture and earthquake hazards in slowly deforming regions. *Geol. Soc. Lond. Sp Pub*. **432**, 1–12 (2017).

[10] Vardar, D., Alp, H., Demirel, S., Vardar, H. A. & Alpar, B. Offshore/onshore correlation of the North-Anatolian fault deformations in the Western sea of Marmara. *Nat. Haz*. **107**, 1905–1923 (2021).

[11] Holmes, J. J., Driscoll, N. W. & Kent, G. M. High-resolution 3D seismic imaging of fault interaction and deformation offshore San Onofre, California. *Front. Earth Sci.* **9**, 653672 (2021).

[12] Moernaut, J. et al. Lacustrine turbidites as a tool for quantitative earthquake reconstruction: new evidence for a variable rupture mode in South central Chile. *J. Geophys. Res. Solid Earth*. **119**, 1607–1633 (2014).

[13] Strasser, M., Anselmetti, F. S., Fäh, D., Giardini, D. & Schnellmann, M. Magnitudes and source areas of large prehistoric Northern alpine earthquakes revealed by slope failures in lakes. *Geology* **34**, 1005–1008 (2005).

[14] Diercks, M-L., Grützner, C., Welte, J. & Ustaszewski, K. Challenges of geomorphologic analysis of active tectonics in a slowly deforming karst landscape (W Slovenia and NE Italy). *Geomorphology* **440**, 108894 (2023).

[15] Altıner, Y. et al. Present day tectonics in and around the Adria plate inferred from GPS measurements. In Dilek, Y., Pavlides, S. (Eds.), Postcollisional tectonics and magmatism in the Mediterranean region and Asia. Geol. Soc. America Sp. Paper **409**, 43–55 (2006).

[16] Moulin, A. et al. The dinaric fault system: Large-scale structure, rates of slip, and Plio-Pleistocene evolution of the transpressive Northeastern boundary of the adria microplate. *Tectonics* **35**, 2258–2292 (2016).

[17] Korbar, T. et al. Active tectonics in the Kvarner region (External Dinarides, Croatia)—An alternative approach based on focused geological Mapping, 3D Seismological, and shallow seismic imaging data. *Front. Earth Sci.* **8**, 582797 (2020).

[18] Atanackov, J. et al. Database of active faults in slovenia: compiling a new active fault database at the junction between the Alps, the dinarides and the Pannonian basin tectonic domains. *Front. Earth Sci.* **9**, 604388 (2021).

[19] Grützner, C. et al. Holocene surface rupturing earthquakes on the dinaric fault System, Western Slovenia. *Solid Earth*. **12**, 2211–2234 (2021).

[20] Picha, F. J. Late orogenic strike-slip faulting and escape tectonics in frontal Dinarides-Hellenides, Croatia, Yugoslavia, Albania and Greece. *AAPG Bull.* **86**, 1659–1671 (2002).

[21] Vičič, B., Aoudia, A., Javed, F., Foroutan, M. & Costa, G. Geometry and mechanics of the active fault system in Western Slovenia. *Geophys. J. Int.* **217**, 1755–1766 (2019).

[22] Rajh, G., Stipčević, J., Živčić, M., Herak, M. & Gosar, A. *& the AlpArray Working Group. Investigation of the Upper Crustal Structure in the NW Dinarides Using Local Earthquake Tomography* (ESS Open Archive, 2024).

[23] Kapuralić, J., Šumanovac, F. & Markušić, S. Crustal structure of the Northern dinarides and Southwestern part of the Pannonian basin inferred from local earthquake tomography. *Swiss J. Geosci.* **112**, 181–198 (2019).

[24] Korbar, T. Orogenic evolution of the external dinarides in the NE Adriatic region: a model constrained by tectonostratigraphy of upper cretaceous to paleogene carbonates. *Earth-Sci. Rev.* **96**, 296–312 (2009).

[25] Kastelic, V. & Carafa, M. M. C. Fault slip rates for the active external dinarides thrust-and-fold belt. *Tectonics* **31**, TC3019 (2012).

[26] Juračić, M., Benac, Č. & Crmarić, R. Seabed and surface sediments map of the Kvarner Bay, Adriatic Sea, Croatia (Lithological map, M 1:500,000). *Geol. Croatica*. **52**, 131–140 (1999).

[27] Brunović, D. et al. High-resolution seismic record of the quaternary palaeoenvironments along a Dalmatian-type Coast (Lošinj Channel, Adriatic Sea). *Mar. Geol.* **474**, 107325 (2024).

[28] Kastelic, V. et al. Seismogenic sources in the Adriatic domain. *Mar. Petrol. Geol.* **42**, 191–213 (2013).

[29] Schmid, S. M. et al. The Alpine-Carpathian-Dinaridic orogenic system: correlation and evolution of tectonic units. *Swiss J. Geosc*. **101**, 139–183 (2008).

[30] Balling, P., Tomljenović, B., Schmid, S. M. & Ustaszewski, K. Contrasting along-strike deformation styles in the central external dinarides assessed by balanced cross-sections: implications for the tectonic evolution of its paleogene flexural foreland basin system. *Gl Plan. Change*. **205**, 103587 (2021).

[31] Špelić, M., del Ben, A. & Petrinjak, K. Structural setting and geodynamics of the Kvarner area (Northern Adriatic). *Mar. Petrol. Geol.* **125**, 104857 (2021).

[32] Kamenski, A. & Korbar, T. Platform-to-Basin evolution of a tectonically indistinct part of a multiple Foreland—Analysis of a 3D seismic block in the Northern Adriatic sea (Croatian Offshore). *Geosciences* **13**, 323 (2023).

[33] Palenik, D. et al. Geological and structural setting of the Vinodol Valley (NW Adriatic, Croatia): insights into its tectonic evolution based on structural investigations. *Geol. Croatica*. **72**, 179–193 (2019).

[34] Benac, Č. & Juračić, M. Geomorphological indicators of the sea-level changes during upper pleistocene (Wuerm) and holocene in the Kvarner region. *Acta Geogr. Croat*. **33**, 27–45 (1998).

[35] Benac, Č., Juračić, M. & Bakran-Petricioli, T. Submerged tidal notches in the Rijeka Bay NE Adriatic sea: indicators of relative sea-level change and of recent tectonic movements. *Mar. Geol.* **212**, 21–33 (2004).

[36] Benac, Č., Bočić, N. & Juračić, M. Geomorphologic changes of the velebit channel during late pleistocene and holocene (NE Adriatic). *Geogr. Fis. Dinam Quat*. **45**, 41–54 (2022).

[37] Dewey, J. F., Holdsworth, R. E. & Strachan, R. A. Transpression and transtension zones. *Geol. Soc. Lond. Sp Publ*. **135**, 1–14 (1998).

[38] Viola, G., Odonne, F. & Mancktelow, N. S. Analogue modelling of reverse fault reactivation in strike–slip and transpressive regimes: application to the Giudicarie fault system, Italian Eastern Alps, *J. Str. Geol.* **26**, 401–418 (2004). (2004).

[39] Herak, M., Herak, D. & Markušić, S. Revision of the earthquake catalogue and seismicity of Croatia, 1908–1992. *Terra Nova*. **8**, 86–94 (1996).

[40] Korbar, T. Conflicting tectonic interpretations of the central external dinarides. *Geol. Croatica*. **78/3**, 257–265 (2025).

[41] Liu, Y. et al. Strike-slip fault zone architecture and its effect on fluid migration in deep-seated strata: insights from the central Tarim basin. *Basin Res.* **36**, e12868 (2024).

[42] Cunningham, W. D. & Mann, P. Tectonics of strike-slip restraining and releasing bends. *Geol. Soc., London, Sp. Pub*. **290**, 1–12 (2007).

[43] Markušić, S., Stanko, D., Korbar, T. & Sović, I. Estimation of near-surface Attenuation in the tectonically complex contact area of the Northwestern external dinarides and the Adriatic foreland. *Nat. Hazards Earth Syst. Sci.* **19**, 2701–2714 (2019).

[44] Bondár, I., Myers, S. C., Engdhal, E. R. & Bergman, E. A. Epicentre accuracy based on seismic network criteria. *Geophys. J. Int.* **156**, 4830496 (2004).

[45] Winton, T. Quantifying Depth of Burial and Composition of Shallow Buried Archaeological Material: Integrated Sub-bottom Profiling and 3D Survey Approaches, in: McCarthy, J.K., Benjamin, J., Winton, T. van Duivenvoorde W., 3D Recording and Interpretation for Maritime Archaeology Springer Coastal Research Library **31**, 154–174, (2020). 10.1007/978-3-030

[46] Wunderlich, J. Mobile parametric sub-bottom profiler systems for shallow and medium depth applications. *J. Acoust. Soc. Am.* **122/5**, 2983 (2007).

[47] Daxer, C. et al. Morphology and spatio-temporal distribution of lacustrine mass-transport deposits in Wörthersee, Eastern Alps, Austria. *Geol. Soc. Lond. Special Publications*. 10.1144/SP500-2019-179 (2019).

[48] Wang, F. et al. An experiment of the actual vertical resolution of the Sub-bottom profiler in an anechoic tank. *Archives Acoust.* **44** (1), 185–194. 10.24425/aoa.2019.126364 (2019).

[49] Unnithan, V. & Rossi, A. P. Enigmatic holocene sand ridges: complex meandering to anastomosing bedforms in the North sea (GermanSeabight). *Geo-MarineLetters* **38**, 417–428. 10.1007/s00367-018-0543-9 (2018).

[50] Yutsis, V., Krivosheya, K., Levchenko, O., Lowag, J. & de León Gómez, H. &Tamez Ponce, A. Bottom topography, recent sedimentation and water volume of the Cerro Prieto Dam, NE Mexico. *Geofísica Int.* **53** (1), 27–38 (2014).

[51] Missiaen, T., Slob, E. & Donselaar, M. E. Comparing different shallow geophysical methods in a tidal estuary, Verdronken land Van Saeftinge, Western Scheldt, the Netherlands. *Neth. J. Geosci. — Geologie En Mijnbouw*. **87** (2), 151–164 (2008).

[52] Wunderlich, J. & Müller, S. High-resolution sub-bottom profiling using parametric acoustics. *Int. Ocean. Syst.* **7** (4), 6–11 (2003).

[53] Trobec, A., Šmuc, A., Poglajen, S. & Vrabec, M. Submerged and buried pleistocene river channels in the Gulf of Trieste (Northern Adriatic Sea): Geomorphic, stratigraphic and tectonic inferences. *Geomorphology* **286**, 110–120. 10.1016/j.geomorph.2017.03.012 (2017).

[54] Novak, A., Šmuc, A., Poglajen, S. & Vrabec, M. Linking the high-resolution acoustic and sedimentary facies of a transgressed late quaternary alluvial plain (Gulf of Trieste, Northern Adriatic). *Mar. Geol.* **419**, 106061. 10.1016/j.margeo.2019.106061 (2020).

[55] Wells, D. L. & Coppersmith, K. J. New empirical relationships among magnitude, rupture length, rupture width, rupture area, and surface displacement. *Bull. Seismological Soc. Am.* **84** (4), 974–1002 (1994).

