# Supplementary figures and images for: Direct evidence of active tectonics along the offshore sector of the Dinaric Fault System
Source: Sci Rep. 2025 Dec 19;16:2442. doi: 10.1038/s41598-025-32243-z (PMC12820375; doi:10.1038/s41598-025-32243-z)

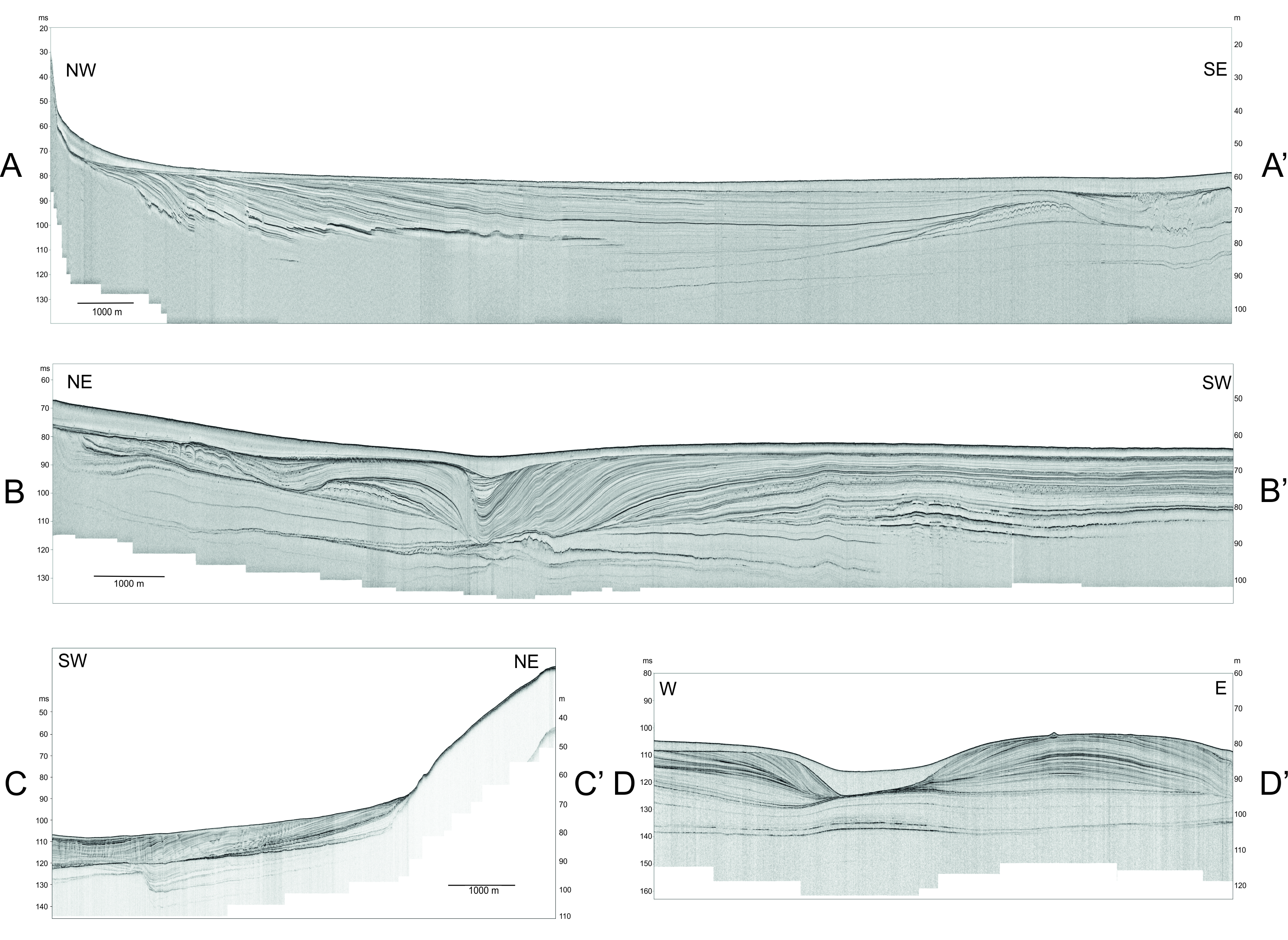

Supplement: Supplementary file 2 — Supplementary Material 2 [file 41598_2025_32243_MOESM2_ESM.jpg]

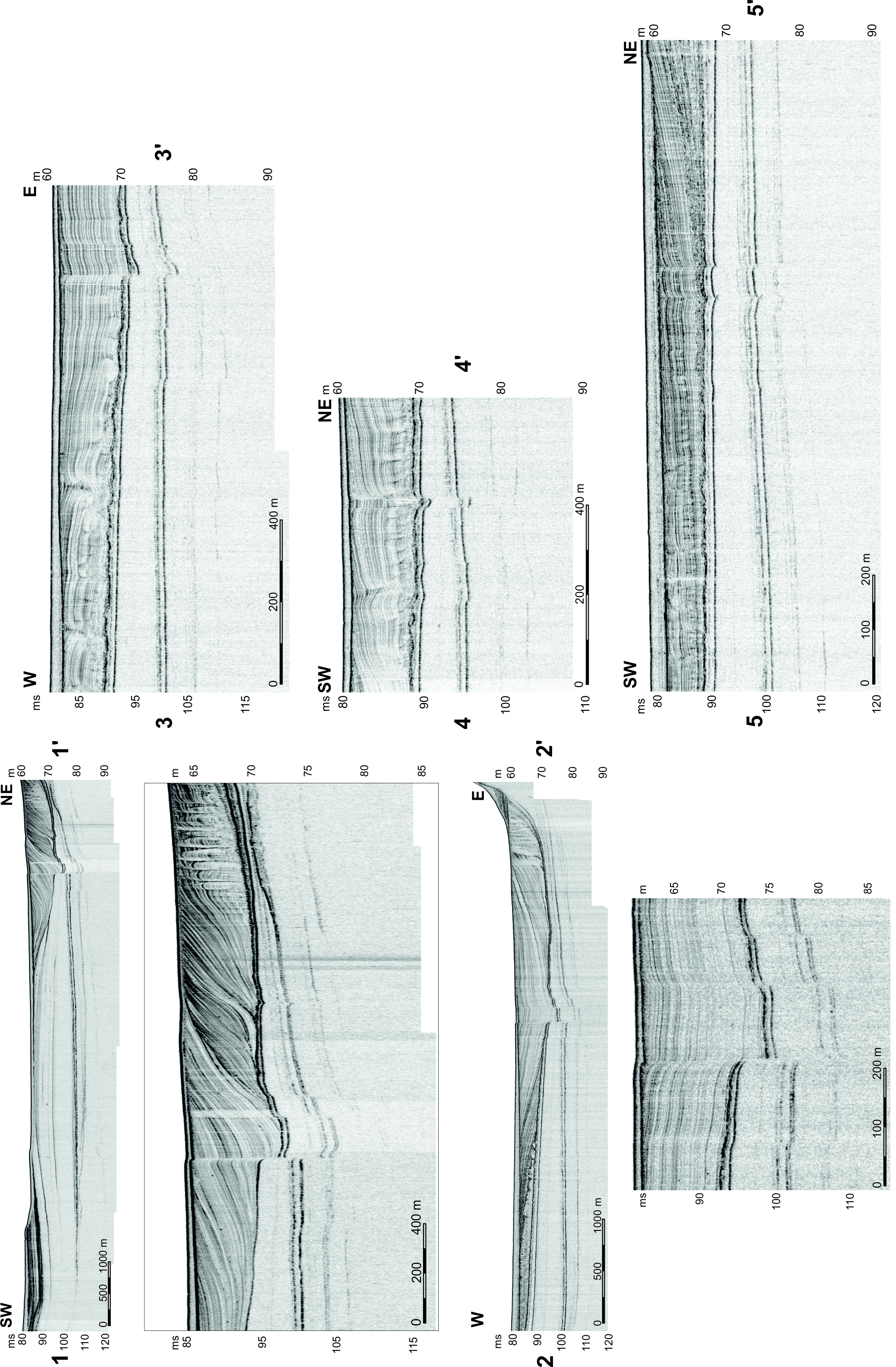

Supplement: Supplementary file 3 — Supplementary Material 3 [file 41598_2025_32243_MOESM3_ESM.jpg]

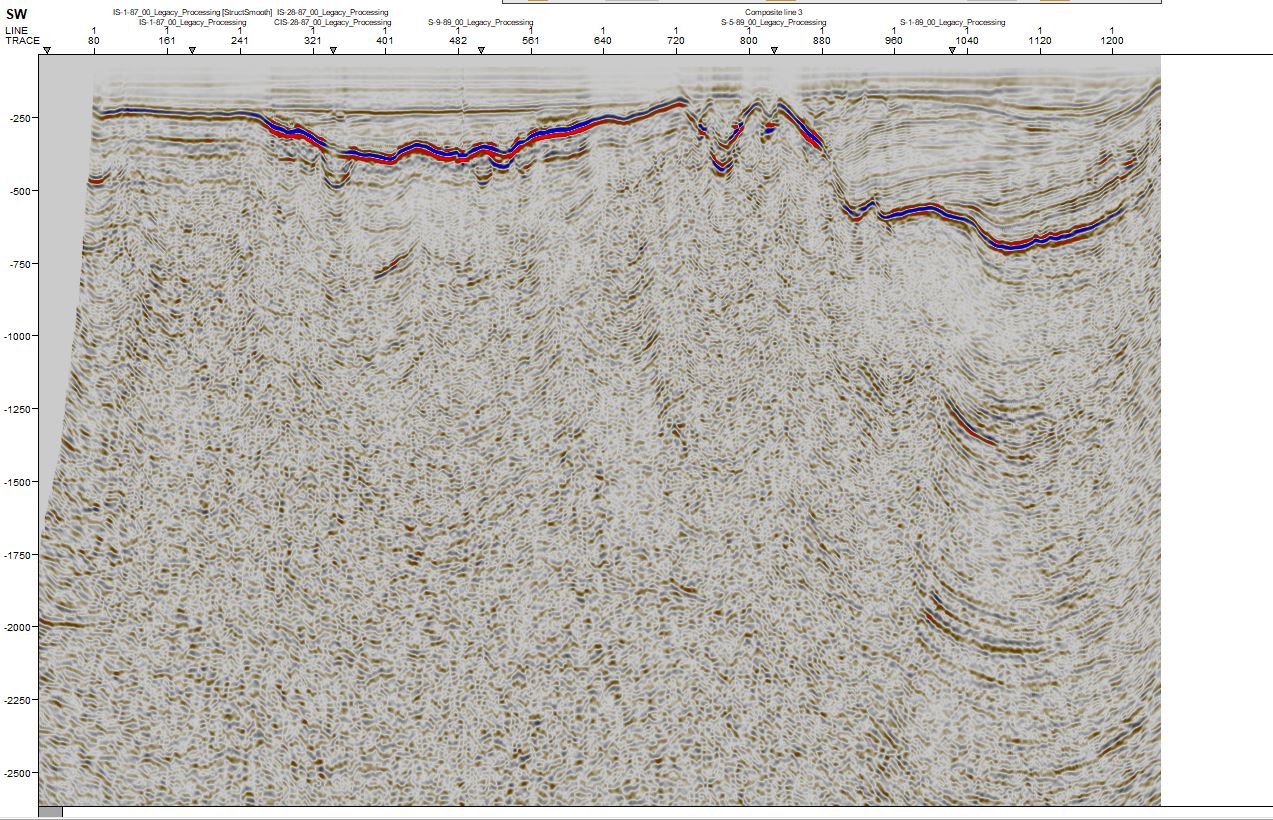

Supplement: Supplementary file 4 — Supplementary Material 4 [file 41598_2025_32243_MOESM4_ESM.jpg]
