# Supplementary material for: Direct evidence of active tectonics along the offshore sector of the Dinaric Fault System
Source: Sci Rep. 2025 Dec 19;16:2442. doi: 10.1038/s41598-025-32243-z (PMC12820375; doi:10.1038/s41598-025-32243-z)
Supplement: Supplementary file 5 — Supplementary Material 5 [file 41598_2025_32243_MOESM5_ESM.docx]

Supplementary file 1. Data table on earthquakes in the wider Kvarner area (M>3.5R) obtained from the Croatian Earthquake Catalogue (CEC). Complete seismological data from the catalogue (CEC) is available for scientific purpose upon written request to the Department of Geophysics, Faculty of Science, University of Zagreb.

Supplementary file 2. Uninterpreted high-resolution seismic data depicted in Fig. 3.

Supplementary file 3. Uninterpreted high-resolution seismic data depicted in Fig. 5.

Supplementary file 4. Uninterpreted deep-seismic profile depicted in Fig. 7c.
